# Reply: Is Epstein–Barr virus associated with aggressive forms of breast cancer?

**DOI:** 10.1038/bjc.2011.98

**Published:** 2011-03-29

**Authors:** C Mazouni, F Fina, P-M Martin

**Affiliations:** 1 Laboratoire de transfert d’oncologie biologique, Assistance Publique – Hopitaux de Marseille, Faculte de Medecine Nord, Bd. Pierre Dramard 13344 Marseille Cedex 15, Marseille, France; 2 Departement de chirurgie generale, Institut Gustave Roussy, 114 rue Edouard Vaillant, Villejuif 94805, France

Sir,

We thank Khan *et al* (2011) for their valuable and insightful comments on our study (Mazouni *et al*, 2011) expounded in their Letter to the Editor (Khan *et al*, 2011).

In our initial publication, we demonstrated the presence of the Epstein–Barr virus (EBV) in malignant breast tumours, which is consistent with findings in previous international publications (Fina *et al*, 2001). In the paper, they are referring to, we observed the same positivity rate as previously noted using a more precise PCR technology. Khan *et al* (2011) suggest that the positivity findings in BC specimens may have been biased by EBV-infected lymphocytes. As mentioned in our paper, our aim was to ascertain the presence of EBV in epithelial cells by isolating these cells using laser microdissection capture (LMC) (Mazouni *et al*, 2011). A large amount of tissue (100 mg) was used to extract DNA from our specimens and this might explain why EBV was detected at a higher frequency than in other studies. In their study, Khan *et al* (2010) did not perform LMC to ensure the validity of epithelial cell positivity.

Besides, another valuable criticism leveled by Khan *et al* (2010) concerns the controversial association between EBV and BC. Other retroviruses have been reported to induce BC. In a previous report, Berebbi *et al* (1990) induced the development of BC in an experimental model by modulating the presence of the polyoma virus during the development of the mammary gland . Moreover, the relationship between EBV and BC has been suggested in an epidemiological study (Yasui *et al*, 2001).

The aggressive profile of EBV-positive BC that we observed has previously been reported in other series (Bonnet *et al*, 1999; Murray *et al*, 2003). Moreover, the differentiation markers evaluated in the breast specimens are associated with the epithelial component.

Finally, we agree that EBV is a ubiquitous infection that makes the physiopathology of BC development difficult to explain. There is still a long road ahead in the field of virus-related cancer before we will be able to formally assess their role and propose prevention.
